# Utilizing the Short Mood and Feelings Questionnaire to measure symptoms of depression among Vietnamese adolescents in Hanoi, Vietnam, during the COVID-19 pandemic

**DOI:** 10.3389/fpsyt.2024.1400128

**Published:** 2024-06-13

**Authors:** Ngo Anh Vinh, Nguyen Thanh Long, Do Thi Trang, Le Thu Trang, Le Thi Thanh Thuy

**Affiliations:** ^1^ Department of Adolescent Health, Vietnam National Children’s Hospital, Hanoi, Vietnam; ^2^ Department of Psychiatry, Hanoi Medical University, Hanoi, Vietnam; ^3^ Binh Minh Center of Education Psychology Research and Application, Hanoi, Vietnam; ^4^ Social Work Faculty, Vietnam Youth Academy, Hanoi, Vietnam

**Keywords:** Short Mood and Feelings Questionnaire, SMFQ, depressive symptoms, adolescent, children, COVID-19

## Abstract

**Objective:**

This study aimed to measure depression among children and adolescents during the COVID-19 pandemic in Hanoi, Vietnam and its associated factors by using the Short Mood and Feelings Questionnaire (SMFQ) instrument.

**Methods:**

We conducted a cross-sectional study among students from grades 6 to 9 within two secondary schools in Hanoi, the capital of Vietnam. A structured questionnaire was used, including information about personal characteristics, perception of COVID-19, and SMFQ. Factor analysis, Multivariate logistic and Tobit regression models were used.

**Results:**

Among 2378 students, 8.8% had depressive symptoms. The mean SMFQ score was 4.5 (SD=5.0). Being female, studying in higher grades, perceived low household income, higher perceived impacts of COVID-19 on health and higher perceived impacts of COVID-19-related quarantine on life were positively associated with factors’ scores, SMFQ score and depressive symptoms. Meanwhile, having better academic performance, living with parents and having higher perceived knowledge about COVID-19 were negatively associated with factors scores, SMFQ score and depressive symptoms.

**Conclusions:**

Depressive symptoms were common among secondary school students in Hanoi, Vietnam, during the COVID-19 pandemic. Tailored interventions to improve pandemic-related knowledge and family and school support should be warranted for the students to enhance their mental well-being.

## 1 Introduction

Mental disorders represent a significant public health concern among adolescents aged 10–19 years (1), and account for 13% of the global burden of diseases and injuries in this population (1). Approximately half of mental disorder cases manifest before the age of 14 and often remain unidentified and untreated (2). These conditions frequently endure for a prolonged duration, causing significant disruptions in adolescents’ ability to obtain livelihoods, access healthcare, and pursue education while also subjecting them to stigmatization, solitude, suicidal tendencies, discriminatory acts, and sexual violence. (3). The mental health disorders of adolescents often persist into adulthood, effectively constraining their potential to lead satisfying lives in their adult years (4). Additionally, it has been observed to be linked to impairments in both social functioning and academic performance, as well as an increased risk of suicide and substance abuse (5). Thus, timely mental disorders identification and treatment among adolescents play a pivotal role in their current and future performance.

COVID-19 is well-known as a threat to the health and social well-being of every affected nation. Despite experiencing a less severe manifestation of COVID-19 in comparison to adults, children and adolescents are adversely affected by the strategies implemented to mitigate the spread of the disease, including the closure of schools and isolation measures. These interventions have had an unfavorable influence on the mental health and overall well-being of children and adolescents (6). A prior literature review suggested that the prevalence rates of depression (7.2%-43.7%), anxiety (15%-78%), and stress (17.3%) were estimated to vary across geographical regions and demographic characteristics (7). The prolonged duration spent on online platforms, the implementation of distance learning, sedentary behavior, insufficient physical activity, feelings of monotony, inadequate sleep, and alterations in dietary patterns have adversely affected the self-perception of children and adolescents. Moreover, these factors have hindered their capacity to partake in age-appropriate developmental tasks while exacerbating their negative emotional states (8, 9). The consequences of the pandemic on children and adolescents include the effects of stress, neglect, and violence within the context of marital and family dynamics, which has raised apprehensions regarding the welfare of parents and other relatives (10).

Although the COVID-19 pandemic has led to an increase in mental disorders among children and adolescents, it has also generated a need for specific actions to minimize the risk of mental disorders in these populations in future pandemics. One significant impediment to timely recognition of potential concerns is the dearth of screening instruments tailored to children and adolescents’ linguistic and everyday experiences. The Short Mood and Feelings Questionnaire (SMFQ) has emerged as a noteworthy choice for accurately evaluating fundamental depressive symptoms in the context of epidemiological research (11). A prior study suggested a robust unidimensional structure and substantial internal consistency of the SMFQ in a study conducted within a population-based sample (12).

In Vietnam, during the COVID-19 pandemic, mental health has been widely investigated in different populations, such as general populations (13), university students (14), or health professionals (15); however, evidence on the mental health of children and adolescents are constrained. Our research question was: What was the rate of depression among adolescents in Vietnam if measured by the SMFQ instrument? What were factors potentially associated with depression among these children and adolescents? This study aimed to measure the depression of children and adolescents in Vietnam by using the SMFQ instrument and evaluate associated factors.

## 2 Materials and methods

### 2.1 Research design

A cross-sectional study was conducted on 6th to 9th-grade students within two secondary schools in Hanoi, the capital of Vietnam. Students, who were currently enrolled in the selected educational institution willingly agreed to participate in the research, and their parents/guardians signed the written informed consent, were invited to participate in the study. A multi-stage sampling procedure was performed. Firstly, we compiled a list of secondary schools in Hanoi, Vietnam. Then, we randomly selected two schools from the list. Finally, all students from these two schools were invited to participate in the research. In total, 2378 students participated in the research. This study was approved by the Institutional Review Board of the National Children’s Hospital.

### 2.2 Data collection method

A structured questionnaire was developed to collect data. The questionnaire was tested on ten students, then revised and approved by school leaders and research groups. During the survey data collection process, the teacher assumed the supervisor role, overseeing the students as they completed the various information entries. To mitigate errors, teachers verified the completeness and accuracy of evaluation forms after collecting them and prompted students to provide any missing or inappropriate information. The survey encompassed personal factors (gender, grade/level, academic performance, children’s living circumstances, awareness of household income), perception of COVID-19 (about impacts on health, impacts of quarantine on daily life, and knowledge about COVID-19), and the Short Mood and Feelings Questionnaire (SMFQ).

Students were asked to rate the impact of COVID-19 on health, the impacts of COVID-19 quarantine on daily life, and their knowledge about COVID-19. The first question was “How would you rate the impact of COVID-19 on your health?” with 11 options from 0 “No impact” to 10 “Extreme impact”. The second question was “How would you rate the impact of COVID-19-related quarantine on your daily life?”, with 11 options from 0 “No impact” to 10 “Extreme impact”. The last question was “How would you rate your knowledge about COVID-19?”, with 11 options from 0 “No knowledge” to 10 “Full knowledge”.

The main focus of this study was to gather information about depressive symptoms in adolescents through the self-reporting approach. The SMFQ instrument, consisting of 13 self-report items, was given to participants to assess their depressive symptoms over the past fortnight. Some instances of items include “I experienced feelings of misery or unhappiness” and “I had a belief that I was no longer valuable”. The items were evaluated using a Likert scale consisting of three points, where 0 represented “Not true” 1 represented “Sometimes” and 2 represented “True”. The potential overall scores ranged from 0 to 26, where higher scores indicated more severe depressive symptoms. The primary outcome of the study was the total score of depressive symptoms. A binary measure was created using the validated cut-off score (12 or higher) to evaluate the influence on caseness prevalence, indicating the presence of significant depressive symptoms (16).

### 2.3 Statistical analysis

The data analysis was done using STATA 15. 0 software (StataCorp LP, College Station, TX, USA). Frequency and % were used to assess qualitative variables, while mean and SD were analyzed for quantitative variables. The exploratory factor analysis (EFA) and principal component analysis (PCA) were used to determine the best structural model for the SMFQ instrument. The scree plot and parallel analysis were used to determine the number of components, eigenvalues, and proportion of variance (17). Items with a loading value ≥ 0.35 are considered relevant. The score for each factor was computed by totaling all factor items and dividing to the number of items in each factor. We used Multilevel Logistic Regression to find factors related to depressive symptoms (Yes=1, No=0). The result was presented in adjusted Odd ratios (aOR) and 95% confidence interval (CI). The Multilevel Tobit Regression model was used to assess associations between characteristics and SMFQ scores and factors’ scores. Level 1 of the model was individuals, and the level 2 was schools. The result was presented in Coefficient (Coef.) and 95%CI. The significance level was reached if p < 0.05.

## 3 Results

Among 2378 students, the proportion of male and female students and students per grade was approximately equal. Most of the students had good study performance (67.70%). Most participants lived with both parents (91.07%) and perceived their household income was moderate (72.39%). According to SMFQ, 8.8% had depressive symptoms, and the mean SMFQ score was 4.46 (SD=5.00). The mean scores of perceived knowledge about COVID-19, perceived impacts of COVID-19 on health and perceived impacts of COVID-19-related quarantine on life were 7.00 (SD=1.97), 4.17 (SD=2.98) and 3.98 (SD=2.98), respectively (**Table 1**).

**Table 1:** Demographic characteristics, SMFQ score and perception related to COVID-19 among students.

Characteristics		Freq. (n)	Percent (%)
**Grade**	6	789	33.18
	7	794	33.39
	≥ 8	795	33.43
**Gender**	Male	1173	49.33
	Female	1205	50.67
**Academic performance**	Very poor	28	1.18
	Poor	176	7.40
	Fair	563	23.72
	Good	1601	67.70
**People living with**	Only father	35	1.48
	Only mother	162	6.86
	Both parents	2152	91.07
	Do not live with parents	14	0.59
**Perceived household income**	High	155	6.58
	Moderate	1704	72.39
	Low	92	3.91
	Do not know	403	17.12
**SMFQ classification**	Not depression (≤ 12)	2168	91.17
	Depression (> 12)	210	8.83
		*M*	*SD*
Perceived knowledge about COVID-19 (0–10)		7.00	1.97
Perceived impacts of COVID-19 on health (0–10)		4.17	2.98
Perceived impacts of COVID-19-related quarantine on life (0–10)		3.98	2.98
SMFQ score (0–26)		4.46	5.04

SMFQ, Short Mood and Feelings Questionnaire.

Results of EFA show a two-factor model when analyzing the SFMQ instrument. The first factor, “Cognitive/Mood”, had eight items, with the value of Cronbach’s alpha = 0.872 and a mean score of 2.17/16 (SD=3.15). The second factor, “Somatic/Emotion”, had five items, with the value of Cronbach’s alpha = 0.763 and the mean score of 2.28/10 (SD=2.31) (**Table 2**).

**Table 2:** Factor loading of SMFQ instrument.

Items		Component		Cronbach’s alpha	*M* (SD)
		1	2		
Somatic/Emotion	3. Tired		0.648	0.763	2.28 (2.31)
	7. Poor concentration		0.600		
	2. Not enjoy		0.584		
	10. Felt lonely		0.476		
	4. Restless		0.417		
Cognitive/Mood	8. Hated self	0.705		0.872	2.17 (3.15)
	9. Bad person	0.696			
	5. Felt no good	0.646			
	13. Everything wrong	0.594			
	11. Unloved	0.567			
	12. Never be as good	0.513			
	6. Cried a lot	0.495			
	1. Miserable	0.476			

**Table 3** shows that the scores of both factors differed across groups regarding grade, gender, academic performance and perceived household income (p<0.05). The score of the “Cognitive/Mood” factor was significantly higher in students who did not live with parents (p<0.05). Meanwhile, no difference was found among groups regarding people living with students.

**Table 3:** Characteristics of two factors according to different groups.

Characteristics	Cognitive/Mood domain				Somatic/Emotion domain			
	*M*	*SD*	*z/χ^2¶^*	p	Mean	SD	*z/χ^2¶^*	p
Grade								
6	1.83	2.74	6.514	0.038**	2.10	2.20	6.593	0.037**
7	2.35	3.32			2.34	2.34		
≥ 8	2.34	3.32			2.40	2.46		
Gender								
Male	1.59	2.57	-8.450	<0.001*	1.89	2.13	-8.450	<0.001*
Female	2.4	3.52			2.66	2.40		
Academic performance								
Very poor	4.77	4.94	27.127	<0.001**	3.00	2.93	8.337	0.040**
Poor	2.80	3.58			2.61	2.55		
Fair	2.43	3.29			2.37	2.23		
Good	1.97	2.97			2.20	2.29		
People living with								
Do not live with parents	5.79	6.61	12.032	0.002**	3.64	4.18	3.880	0.144**
Only the father or mother	2.95	3.75			2.60	2.45		
Both parents	2.08	3.03			2.24	2.28		
Perceived household income								
High	1.86	2.88	19.794	<0.001**	1.94	2.33	12.115	0.007**
Moderate	2.07	3.01			2.29	2.27		
Low	3.99	4.38			3.16	3.01		
Do not know	2.31	3.38			2.18	2.24		

*Mann-Whitney test; **Kruskal-Wallis test; ^¶^z for Mann-Whitney test; χ^2^ for Kruskal-Wallis test.

**Table 4** indicates that in comparison with male students, female students had a significantly higher Cognitive/Mood score (Coef. = 1.11, 95%CI=0.88 - 1.35); Somatic/Emotion score (Coef. = 0.69, 95%CI=0.51–0.86), SMFQ score (Coef. = 1.80; 95%CI = 1.42 - 2.17) and a higher likelihood of having depression (OR=2.85, 95%C=2.04 - 4.00). Similarly, students in grade 7 and grade 8 had a significantly higher Cognitive/Mood score, Somatic/Emotion score, SMFQ score and a higher likelihood of suffering depression than those in grade 6 students.

**Table 4:** Factors associated with SMFQ score, subscales’ scores and depression classification.

Characteristics	Cognitive/Mood score			Somatic/Emotion score			SMFQ score			SMFQ classification (0=No, 1=Depression)		
	Coef.	95%CI		Coef.	95%CI		Coef.	95%CI		aOR	95%CI	
Gender												
Male	ref			ref			ref			ref		
Female	1.11*	0.88	1.35	0.69*	0.51	0.86	1.80*	1.42	2.17	2.85*	2.04	4.00
Grade												
Grade 6	ref			ref			ref			ref		
Grade 7	0.63*	0.35	0.92	0.32*	0.11	0.53	0.95*	0.50	1.40	2.02*	1.35	3.04
Grade 8 or above	0.63*	0.34	0.92	0.39*	0.18	0.60	1.02*	0.57	1.48	2.56*	1.71	3.84
Academic performance												
Very poor	ref			ref			ref			ref		
Poor	-1.21*	-2.39	-0.04	-0.05	-0.91	0.80	-1.26	-3.10	0.58	1.06	0.33	3.37
Fair	-1.41*	-2.54	-0.29	-0.15	-0.97	0.67	-1.55	-3.32	0.21	0.87	0.29	2.65
Good	-1.94*	-3.06	-0.83	-0.35	-1.16	0.46	-2.29*	-4.04	-0.54	0.63	0.21	1.88
People living with												
Not living with both parents	ref			ref			ref			ref		
Living with parents	-0.76*	-1.14	-0.37	-0.21	-0.49	0.07	-0.96*	-1.56	-0.36	0.71	0.47	1.07
Perceived household income												
High	ref			ref			ref			ref		
Moderate	-0.17	-0.65	0.31	0.01	-0.34	0.36	-0.15	-0.90	0.60	0.85	0.42	1.71
Low	1.11*	0.35	1.87	0.43	-0.13	0.99	1.53*	0.33	2.73	1.97	0.81	4.81
Do not know	0.10	-0.44	0.64	-0.07	-0.46	0.32	0.03	-0.81	0.88	1.15	0.53	2.46
**Perceived impacts of COVID-19 on health (0–10)**	0.08*	0.02	0.14	0.07*	0.02	0.11	0.15*	0.05	0.24	1.02	0.95	1.10
**Perceived knowledge about COVID-19 (0–10)**	-0.17*	-0.23	-0.11	-0.16*	-0.21	-0.12	-0.33*	-0.43	-0.23	0.83*	0.77	0.89
**Perceived impacts of COVID-19-related quarantine on life (0–10)**	0.23*	0.17	0.29	0.21*	0.17	0.26	0.44*	0.35	0.54	1.30*	1.21	1.40

Coef., Coefficient; aOR, adjusted Odd ratios; *p<0.05.

Compared to students with very poor performance, students with poor (Coef.=-1.21, 95%CI=-2.39 - -0.04), fair (Coef.=-1.41, 95%CI=-2.54 - -0.29) or good (Coef.=-1.94, 95%CI= -3.06 - -0.83) performance had a significantly lower Cognitive/Mood score. Students with a good performance had a significantly lower SMFQ score (Coef.=-2.29, 95%CI=-4.04 - -0.54) than those with very poor performance.

Students living with parents had a lower Cognitive/Mood score (Coef.=-0.76, 95%CI=-1.14 - -0.37) and SMFQ score (Coef.= - 0.96, 95%CI = -1.56 - -0.36). Meanwhile, in comparison with students who perceived high household income, students perceiving low household income had a higher Cognitive/Mood score (Coef. = 1.11; 95%CI=0.35–1.87) and SMFQ score (Coef. = 1.53, 95%CI=0.33–2.73).

Scores in perceived impacts of COVID-19 on health and perceived impacts of COVID-19-related quarantine on life were positively correlated with Cognitive/Mood score, Somatic/Emotion score, and SMFQ score. Scores in perceived impacts of COVID-19-related quarantine on life were positively related to a higher likelihood of having depression (OR=1.30, 95%CI=1.21–1.40). Scores in perceived knowledge about COVID-19 were negatively correlated with Cognitive/Mood scores, Somatic/Emotion scores, and SMFQ scores and were associated with a lower likelihood of suffering depression.

## 4 Discussion

The deleterious effects of the COVID-19 pandemic are permeating various facets of society, encompassing not only health but also the social realms (18, 19). This study aimed to ascertain the depression of Vietnamese school-aged adolescents with the impacts of COVID-19. Moreover, we found different sociodemographic characteristics and perceptions regarding COVID-19 related to the SMFQ scores.

In light of the global COVID-19 pandemic, the Vietnamese government implemented stringent measures encompassing the closure of educational institutions, the imposition of travel restrictions, the implementation of localized lockdowns, and the enforcement of quarantine protocols within the general populace (20). Control measures are commonly enforced, but variations in intensity may vary across different provinces and between rural and urban areas. This phenomenon may be attributed, at least in part, to the variation in psychological effects observed among students hailing from diverse provinces and residing in different localities. In the present study, 8.8% of students had depressive symptoms according to the SMFQ instrument. This result was in line with a previous study in China, which found that 7.18% of 7–15-year-old children experienced depressive symptoms based on SMFQ assessments (21).

In literature, SMFQ is considered to be unidimensional (22, 23). However, in the present study, based on the results of EFA, we identified two factors, namely Somatic/Emotion and Cognitive/Mood. We differentiate between emotion and mood as they represent distinct affective states (24). An emotion can be described as a transient and intense feeling that is often focused on a particular stimulus. It is common for emotions to be manifested through distinct facial expressions and body language. A mood can be characterized as a mental state of lesser intensity than an emotion, and does not invariably require a situational trigger. The duration of moods exceeds that of emotions, ranging from hours to days (24). It is noteworthy to observe that item 2 “Not enjoy”, item 7 “Poor concentration”, and item 10 “Felt lonely” are classified into the same group as “Tired” and “Restless” to form the “Somatic/Emotion” group. This may be attributed to the symptom of “Poor concentration” as students do not perceive it as a cognitive-related condition but rather as a consequence of being “tired,” thus these two symptoms tend to co-occur. On the other hand, we observed that symptoms such as “Felt lonely” or “Not enjoy” are short-term symptoms and are influenced by social environmental factors as well as somatic symptoms (25); thus, these items tend to co-occur. Meanwhile, the items within the “Cognitive/Mood” factor are cognitive problems or chronic affective states such as “Hated self” or “Unloved” (26). In the present study, we observed that the issues related to Somatic/Emotion and Cognitive/Mood were scored relatively similarly, indicating that students experienced these issues to a comparable extent, suggesting that further interventions should be warranted to address both aspects.

Despite the decreasing trend of the COVID-19 pandemic, its impact remains significant, especially for children previously affected by the pandemic. The fact that depressive symptoms were highly prevalent in children could be expected, considering the significant and unprecedented negative impact that COVID-19 has had on daily lives. Adolescents were restricted to their residences, anxiously concerned about their schooling and apprehensive about the potential of getting infected, which could cause significant financial hardships for their families (27, 28). Moreover, the excessive amount of news coverage and attention-grabbing headlines about the COVID-19 situation have unavoidably caused distress, apprehension, and unease among the general public, including young individuals (27, 28). When integrated, these factors possess the potential to influence the psychological well-being of children significantly. As students’ perceptions regarding the impact of COVID-19 on health and daily life increase, so does their corresponding score on the SMFQ scale. This demonstrates that the COVID-19 pandemic significantly impacts the mental health of adolescents. We also observed that adolescents perceived a greater severity of impact and were more frequently subjected to the consequences of COVID-19, such as the impact on family members’ health or their parents’ employment and educational pursuits. On the other hand, adolescents who self-perceived themselves as having a good knowledge of COVID-19 exhibited lower SMFQ scores, indicating that they had prepared psychologically to cope with the pandemic. This suggests that meticulous education of students about the COVID-19 pandemic or future pandemics plays a significant role in ensuring the mental health conditions of adolescents during times of crisis.

The current study corroborated the findings of multiple prior studies regarding higher scores on measures that assess depressive symptoms in girls compared to boys (29, 30). Numerous studies have underscored the significance of vigilance toward depressive symptoms in girls, as they are associated with potential adversities in both social and academic domains (5). We also observed that students studying in higher grades or, in other words, students of higher age, were more likely to have depressive symptoms, which aligned with prior studies (21, 31, 32). It is noteworthy that there is no significant difference in the prevalence of depressive symptoms between students in grade 7 and those in grade 8 or above. This phenomenon may be attributed to the fact that sixth grade students are transitioning to a new level of education (from elementary to secondary schools) and are not yet familiar with the study methods employed in secondary school. This engenders difficulties in their learning and consequently renders them more susceptible to issues of depression compared to students in higher grades.

In addition, better academic performance was negatively related to Cognitive/Mood scores. Indeed, depression has been observed to correlate significantly with impairments in concentration, social interactions, academic achievement, and reading and writing skills. Additionally, individuals experiencing depression commonly perceive their academic responsibilities as being burdensome (33). Lower household income was also related to Cognitive/Mood scores. In literature, children from socioeconomically disadvantaged backgrounds face a heightened susceptibility to adverse health consequences, encompassing childhood mental disorders as an essential area of concern (34, 35). A prior study discovered that mental disorders exhibited a significantly higher occurrence rate of three to four times in children whose parents belonged to the lowest income percentiles as opposed to children whose parents fell within the highest income percentiles (36). The COVID-19 pandemic might reduce household income (6), which even amplified the negative impacts on the mental health of children. Furthermore, the research findings also demonstrate that students who do not live with their parents have higher cognitive and affective abilities than students who live with their parents. The aetiology of mental health problems is significantly influenced by familial factors, encompassing both life events and the intra-familial environment. This impact is acknowledged to stem from a combination of psychosocial and genetic mechanisms (37). A prior investigation demonstrated a strong association between a diminished degree of perceived parental support and an elevated likelihood of experiencing any form of mental health-related complication (38).

The present study can be credited for its notable strengths, primarily the considerable sample size employed. However, this study is deemed a primary limitation due to the absence of inclusive information on co-occurring issues. In the adolescent population, the prevalence of comorbidity between anxiety and ADHD, alongside other neuro-developmental disorders, is commonly observed and has a significant impact on long-term persistence and functional outcomes (39). Moreover, the current study did not explore potential variances in functional outcomes related to school functioning and leisure time activities. Moreover, we could not identify the cut-off points for the new subscales, given a lack of gold diagnostic standards. Further studies should be warranted to identify the optimal cut-off points of the “Cognitive/Mood” and “Somatic” domains. Finally, the cross-sectional design utilized in the current study inherently restricts the ability to explore and examine developmental trajectories.

This study holds significant practical implications. Interventions aimed at preventing depression among secondary students should prioritize measures designed to enhance understanding of COVID-19 prevention strategies and to address the adverse effects of the pandemic on students’ physical and mental well-being. Amid the onset of the COVID-19 pandemic, numerous nations and urban centers have instituted measures to enforce social distancing. Consequently, the implementation of conventional interventions to ameliorate depression among adolescents has become challenging. Priority should be given to interventions targeting students in the sixth grade, particularly those who do not reside with their parents. It is imperative to ensure that counseling services are made available to these students to enhance their readiness for the prevention of depression. During the COVID-19 pandemic, virtual counseling sessions are being increasingly utilized; nevertheless, in non-pandemic times, in-person counseling sessions should be duly considered. It is recommended that regular screening be conducted to monitor the presence of depression in students, as well as to provide timely and suitable interventions.

## 5 Conclusion

In conclusion, in addition to highlighting the prevalence and associated factors of depression among children and adolescents in Hanoi, Vietnam, amidst the COVID-19 pandemic, this study underscores the multifaceted nature of mental health in the context of crisis. The observed positive associations between depressive symptoms and factors such as gender, grade level, and household income perception suggest the presence of social and economic disparities that may exacerbate psychological distress among vulnerable populations. Furthermore, the significant impact of perceived health and quarantine-related concerns related to COVID-19 underscores the psychological toll of public health measures and the importance of addressing not only the physical but also the mental well-being of young individuals during times of uncertainty.

Conversely, the negative associations identified between depressive symptoms and factors such as academic performance and perceived knowledge about COVID-19 point to potential protective factors that may buffer against the adverse effects of the pandemic on mental health. These findings highlight the importance of fostering resilience and equipping children and adolescents with accurate information and resources to cope effectively with stressors and uncertainties associated with the COVID-19 pandemic.

Overall, this study contributes valuable insights into the complex interplay between individual, social, and environmental factors in shaping mental health outcomes among young people in the context of a global health crisis. By identifying both risk and protective factors, these findings inform targeted interventions and support efforts to promote the mental well-being of children and adolescents in Hanoi, Vietnam, and beyond, during and beyond the COVID-19 pandemic.

## Data availability statement

The raw data supporting the conclusions of this article will be made available by the authors, without undue reservation.

## Ethics statement

The studies involving humans were approved by the Institutional Review Board of the National Children’s Hospital. The studies were conducted in accordance with the local legislation and institutional requirements. Written informed consent for participation in this study was provided by the participants’ legal guardians/next of kin.

## Author contributions

NV: Conceptualization, Investigation, Methodology, Writing – original draft, Writing – review & editing. NL: Formal analysis, Investigation, Methodology, Supervision, Writing – original draft, Writing – review & editing. DT: Formal analysis, Resources, Supervision, Writing – original draft, Writing – review & editing. LTT: Investigation, Project administration, Validation, Visualization, Writing – original draft, Writing – review & editing. LTTT: Project administration, Resources, Validation, Visualization, Writing – original draft, Writing – review & editing.
